# Supplementary material for: The microbiome biomarkers of pregnant women’s vaginal area predict preterm prelabor rupture in Western China
Source: Front Cell Infect Microbiol. 2024 Oct 31;14:1471027. doi: 10.3389/fcimb.2024.1471027 (PMC11560878; doi:10.3389/fcimb.2024.1471027)
Supplement: Supplementary file 1 [file DataSheet1.zip › compare_1/Community/Pieplot/species/sample/P35.species.pieplot.pdf]

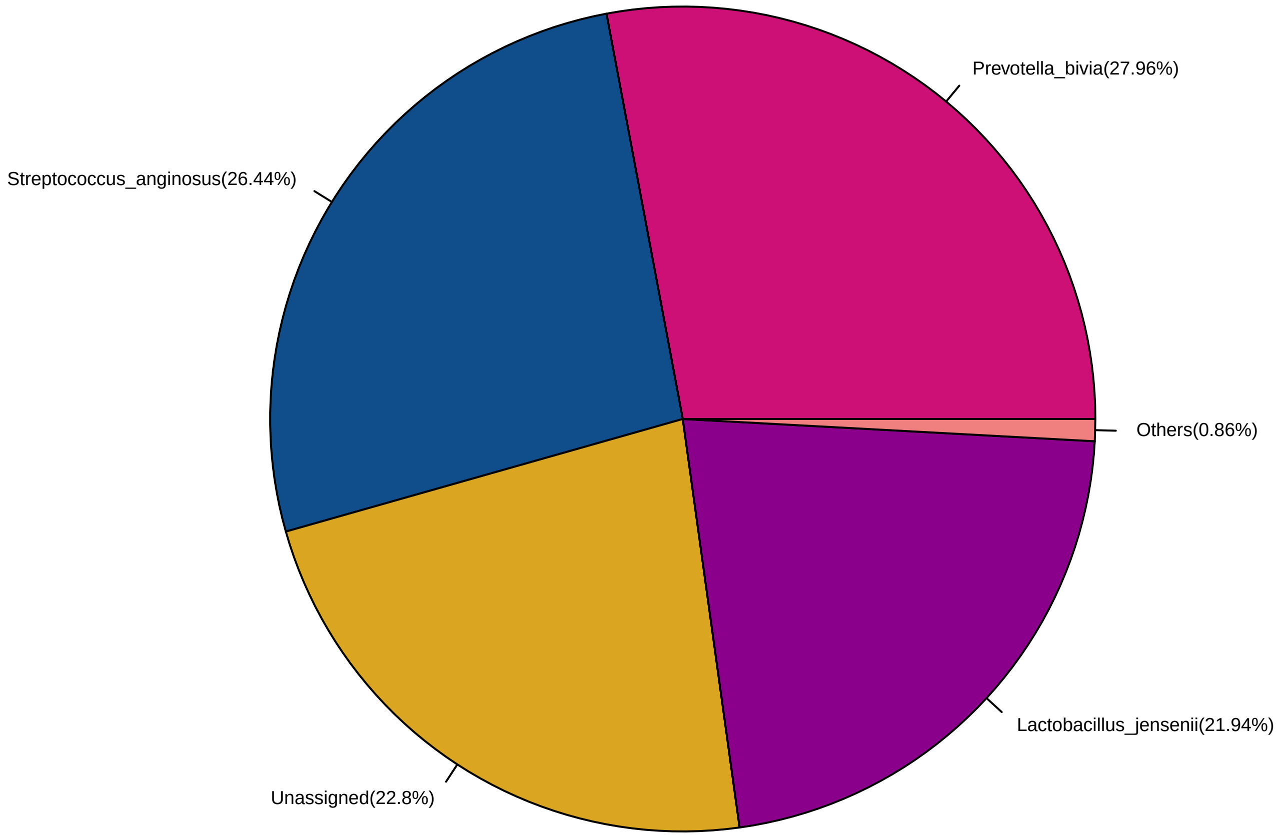
Prevotella_bivia(27.96%)
Streptococcus_anginosus(26.44%)
Others(0.86%)
Lactobacillus_jensenii(21.94%)
Unassigned(22.8%)
